# Annotations

**Published:** 1904-10-22

**Authors:** 


### Annotations.

### An Expert's Opinions.

It is lamented by many who are interested in pathological science and in the advance of the medical art, that in so few instances is the cause of death verified by post-mortem examination. Apart from the educational value of such examinations to the individual practitioner, it is recognised that were the practice more common, the Registrar-General's returns would acquire a precision and significance which in existing circumstances they can hardly be said to enjoy. This view is based upon the supposition that a post-mortem examination would afford a definite and scientific interpretation of the actual cause of death in each individual case, and would thus contribute to the collection of information of great medical and social value. Judging from some recent incidents reported in the public prints, however, there is reason to doubt whether this expectation would be fully realised. The events to which reference is now made may be found in the accounts of two inquests conducted by the Coroner for the City of Westminster. At each of these there appeared as a witness one and the same medical man, and on both occasions this gentleman as a result of a post-mortem examination is reported to have expressed the opinion that death was due to "heart failure from convulsions." Further, in one case the child was said to have suffered from "irritation" caused by improper feeding, and in the other to have been "killed slowly" by the "family feeding it on what they ate themselves." These are truly remarkable discoveries to make in the course of an examination of a dead body, and certainly suggest possibilities not hitherto contemplated by scientific pathology. That "convulsions," "irritation," and the scheme of the family dietary can be detected in this manner is a startling proposition, which, if not true, is at least novel. Expert evidence has in its day had many hard things said of it, but if this latest development of its pretensions is to be continued, it may expect to be killed by ridicule. The amusing aspect of the matter is that the "opinions" above related are received by a coroner who has rendered himself conspicuous by his demands for expert medical testimony, and they are served up to him by the "pathologist" whom he specially delights to honour.

### The Multiplication of Unhealthy Flats.

At the annual meeting of the Society of Medical Officers of Health the new president, Dr. J. F. Sykes, observed that in the centres of our large cities flats for all classes appear to be a modern necessity. He saw no reason why none but healthy flats should be constructed, but he was obliged to say that we had not reached that stage yet. It is to be feared that we are yet a great way off. Many modern flats are quite as insanitary as houses which have been condemned as unfit for habitation, and they will continue to be so until the fundamental error of administration on which Dr. Sykes lays his finger is rectified. He rightly contends that, instead of the medical officer being called upon to certify after construction, he should be afforded the opportunity of advising before, and of suggesting improvement and simplification. This proposal is made not only in the interests of health but also in those of economy. There is no doubt, as Dr. Sykes suggests, that under the present system considerable expense is incurred which might easily be avoided. If, at the outset, flats are perfectly sanitary, ordinary attention will suffice to keep them so. But it is inevitable that many flats which are built without consulting experts sooner or later will have to be altered, and a more or less serious outlay on the part of the owner thus becomes essential. Our interest in the question is, however, in relation to the preservation of the public health and the prevention of physical deterioration. To lock the stable door after the steed has gone is always a stupid policy; when it is pursued in respect to issues of life and death it is insensate folly. Flats, as a fashion, were imported from America, and though we are not sure that their importation has been on the whole as conducive to the welfare of the community as some imagine, their convenience is undeniable. At any rate, they have come to stay, and it is the duty of all who are entrusted with the guardianship of health to see that they are not fertile breeding-grounds of disease. But the multiplication of unhealthy flats can only be arrested by rendering it compulsory for builders not to proceed with the erection of blocks of flats until their plans have been approved by the accredited medical representative of the local authorities.

### The Medico-Legal Society.

This society, which has now entered on its second session, is exhibiting a commendable degree of activity and enterprise. The subjects included within its programme are of importance, not only to members of the legal and medical professions, but also to many departments of municipal and national polity. Hence we are glad to see that its membership contains representatives of those concerned in the administrative activities of public life. It is much to be desired that contributions from such sources shall be made to the discussion of subjects that touch equally the professions of law and medicine. Another evidence of the foresight and wisdom exhibited by the officials of the Medico-Legal Society is the early and prompt publication of a volume of Transactions which contains a number of highly interesting and valuable articles. In the place of honour is the inaugural address of the President, Sir William Collins, who, in philosophic and dignified language, defines the territory the society has to explore and explains the necessity for a special organisation to undertake this duty. From the medical side there are papers on such subjects as medico-legal post-mortem examinations, abortion by drugs, and punctured wounds of the heart. A paper on "Medical Evidence" is contributed by Earl Russell and a note on the Whitechapel murders of 1888 by Mr. F. Gordon Brown, whilst Dr. Wynn Westcott writes on the overlaying of infants. For the new session Sir William Collins has been re-elected president and Sir Thomas Stevenson a vice-president. A programme of much interest has already been arranged, and this, amongst other items, includes a paper on "Evidence," by Sir Joseph Walton, one of his Majesty's judges. Those wishing to join the society should communicate with Dr. Stanley B. Atkinson, 22 Albermarle Street, W.

